# Characterisation of New *Foxunavirus* Phage Murka with the Potential of *Xanthomonas campestris* pv. *campestris* Control

**DOI:** 10.3390/v16020198

**Published:** 2024-01-27

**Authors:** Peter V. Evseev, Rashit I. Tarakanov, Ha T. N. Vo, Natalia E. Suzina, Anna A. Vasilyeva, Alexander N. Ignatov, Konstantin A. Miroshnikov, Fevzi S.-U. Dzhalilov

**Affiliations:** 1 Department of Plant Protection, Russian State Agrarian University—Moscow Timiryazev Agricultural Academy, Timiryazevskaya Str. 49, 127434 Moscow, Russia; petevseev@gmail.com (P.V.E.); annadacyk64@gmail.com (A.A.V.); an.ignatov@gmail.com (A.N.I.); kmi@bk.ru (K.A.M.); dzhalilov@rgau-msha.ru (F.S.-U.D.); 2 Shemyakin-Ovchinnikov Institute of Bioorganic Chemistry, Russian Academy of Sciences, Miklukho-Maklaya Str. 16/10, 117997 Moscow, Russia; 3 Laboratory of Molecular Microbiology, Pirogov Russian National Research Medical University, Ostrovityanova 1, 117997 Moscow, Russia; 4 Faculty of Agronomy, Nong Lam University, Quarter 6, Thu Duc District, Ho Chi Minh City 721400, Vietnam; ha.vothingoc@hcmuaf.edu.vn; 5 Skryabin Institute of Biochemistry and Physiology of Microorganisms, Federal Research Center “Pushchino Center for Biological Research of the Russian Academy of Sciences”, Prosp. Nauki, 5, 142290 Pushchino, Russia; suzina_nataliya@rambler.ru; 6 Agrobiotechnology Department, Agrarian and Technological Institute, RUDN University, Miklukho-Maklaya Str., 6, 117198 Moscow, Russia

**Keywords:** *Xanthomonas* bacteriophages, cabbage, *Brassicae*, biocontrol, bacteriophage taxonomy

## Abstract

Phages of phytopathogenic bacteria are considered to be promising agents for the biological control of bacterial diseases in plants. This paper reports on the isolation and characterisation of a new *Xanthomonas campestris* pv. *campestris* phage, Murka. Phage morphology and basic kinetic characteristics of the infection were determined, and a phylogenomic analysis was performed. The phage was able to lyse a reasonably broad range (64%, 9 of the 14 of the *Xanthomonas campestris* pv. *campestris* strains used in the study) of circulating strains of the cabbage black rot pathogen. This lytic myovirus has a DNA genome of 44,044 bp and contains 83 predicted genes. Taxonomically, it belongs to the genus *Foxunavirus*. This bacteriophage is promising for use as a possible means of biological control of cabbage black rot.

## 1. Introduction

Cabbage (*Brassica oleracea* var. *capitata* L.) is one of the main vegetable crops in the world, due to its nutritional properties and benefits for humans [1]. Russia is the third largest producer of cabbage in the world, with a gross annual harvest of about three million tons [2]. Black rot caused by the Gram-negative bacterium *Xanthomonas campestris* pv. *campestris* (hereinafter, Xcc) is one of the most destructive diseases of this crop [3]. The pathogen is transmitted mostly by latent seed infection [4,5], can be spread by wind, precipitation, and insects [6], and can survive in plant debris. Xcc is one of the “Top 10” most important bacterial pathogens of plants [7].

Pathogen cells penetrate the leaves through hydathodes, stomata, or mechanical wounds, and spread through the vascular system of the plant. Xcc has a polar flagellum, which allows the bacterium to move through the vascular system [8]. Symptoms include the necrotic darkening of leaf veins and V-shaped chlorotic lesions [9,10]. The disease causes premature leaf fall, stunting, and even the death of plants. Xcc is particularly dangerous, being difficult to control due to its numerous virulence factors.

Black rot drastically reduces the yield and quality of cabbage crops. Xcc is regarded as an economically significant pathogen in many countries, including Brazil, Ethiopia, South Africa, Belgium, Germany, Sweden, France, the Netherlands, Italy, the USA, the UK, Nepal, China, Canada, Australia, India, and Russia [11]. To date, 11 pathogenic Xcc races that affect plants of the *Brassicae* family have been identified [12,13]. Worldwide dissemination of the most aggressive strains significantly reduces the efficiency of plant breeding for resistance to this pathogen [14].

Numerous approaches are used to detect and prevent the spread of Xcc in cabbage crops. Early diagnostics of the pathogen are provided by PCR-based assays [15]. A selection of Xcc-resistant hybrids of brassicas is promising, but the results are ambiguous [16]. The prevailing method to control the pathogen is the use of chemicals, in particular, copper-based pesticides [17] and antibiotics [18]. This approach is limited by the harmful effects of copper on products, humans, and the environment, and the rapid evolution of antibiotic-resistant strains of Xcc. The biological approach is based on antagonistic bacteria [19] and botanical pesticides [20], which usually have a weak biological effect on the disease [21]. Given the current situation, the search for new strategies for the reduction of cabbage black rot is an urgent task.

The prospects of bacteriophages (bacterial viruses) as agents for combatting bacterial diseases of plants, including cabbage black rot, have been considered [22,23]. Their application constitutes a promising multi-faceted approach to controlling the bacterial pathogens of plants, to increase the yield of agricultural crops and promote crop preservation [24]. The favourable, or zero, influence of phages on eukaryotes, host specificity, self-replication, and the simplicity of biosynthesis have all aroused interest in them as agents for biological control [25]. Their ubiquity and abundance in the biosphere make it possible to isolate them from environmental sources.

The modern concept of phage therapy suggests that a thorough characterisation of the phage is necessary to enable the scientifically proven use of phages, including in-depth studies of the genome and features of its biology [25,26,27]. To increase the effectiveness of phage treatment, the use of a “phage cocktail” is recommended. A set of phages in the same formulation should be capable of lysing most of the pathogen population. An ample collection of phages different in taxonomic attributions and isolated in diverse geographical locations is needed for this purpose. In this regard, the aim of the study was to characterise a new bacteriophage Murka, active against *Xanthomonas campestris* pv. *campestris* strains.

## 2. Materials and Methods

### 2.1. Bacterial Strains and Their Cultivation

#### 2.1.1. Isolation of Strains

The strains assigned to Xcc were isolated from cruciferous plants with visual signs of black rot (cauliflower and white cabbage from Russia, Moldova, Ukraine, Belarus, and the Netherlands) and shepherd’s purse plants (from Japan). The surface of the stems and shoots were disinfected with ethanol; necrotic vessels were cut out, crushed in sterile water, vortexed, and spread over the surface of YDC medium plates (g/L: yeast extract—10.0, calcium carbonate (CaCO_3_)—20.0, agar—15.0, anhydrous glucose—20.0) [28]. Petri dishes were incubated at 26 °C for 48 h. Yellow-pigmented mucoid colonies, typical for Xcc, were picked and 3-fold recultivation was carried out to obtain a pure culture. For the primary characterisation of the strains, a starch hydrolysis test was performed according to [29], with modifications. Bacterial isolates were applied, in strips, to Petri dishes with nutrient agar containing 0.2% soluble starch (*v*/*v*) and incubated at 30 °C until intensive growth occurred. Then, the dishes were filled with IKI solution (iodine—1 g; potassium iodide—2 g; distilled water—100 mL) and transparent zones indicating starch hydrolysis around the colonies were identified as a positive reaction. Strain 528T from the NCPPB collection (the National Collection of Plant Pathogenic Bacteria, York, UK) was also used as a reference strain. Bacterial cultures were stored in 15% glycerol at −72 °C for subsequent tests. The bacterial strains used in this study are listed in Table 1.

#### 2.1.2. Checking Bacterial Pathogenicity 

A pathogenicity test was performed according to [30], with modifications. Plants of cauliflower cv. Garantiya (susceptible standard) were grown in a winter glazed greenhouse at 28/22 °C (14 h day/10 h night), with natural insolation and watering as needed. The plants were cultivated in a peat–perlite substrate (Veltorf, Velikiye Luki, Russia) in 40-cell plastic trays (cell volume 0.12 L, AgrofloraPack, Vologda, Russia) until phase 3–4 leaves were produced. Inoculation was performed by pricking the leaf vein with a needle dipped in a bacterial suspension in water at a concentration of ~10^9^ CFU (colony-forming units)/mL. Suspensions were prepared from colonies of strains grown on King’s B medium at 26 °C for 48 h, further suspended in sterile water, and adjusted to the optimal concentration measuring the optical density at 600 nm using a NanoDrop One spectrophotometer (Thermo Fisher Scientific, Waltham, MA, USA) in OD_600_ analysis mode. A suspension of NCPPB 528T strain was used as a positive control and sterile water was used as a negative control. Each strain was inoculated on 3 plants. The examination and registration of symptoms were carried out on the 12th day after inoculation. The strains that presented typical symptoms of black rot were used.

#### 2.1.3. Genetic Identification of Xcc Strains

DNA was isolated from two-day liquid cultures using a “Fitosorb” DNA extraction kit (Syntol, Moscow, Russia), according to the manufacturer’s protocol. Reaction mixtures containing 5 µL of 5×Master-mix (5×MasDDTaqMIX-2025, Dialat, Moscow, Russia), 10 pM of each primer, and 5 ng of the target DNA (25 µL total) were used for PCR amplification in a Thermocycler T100 (Bio-Rad, Hercules, CA, USA) [31]. Primers rD1 and fD1 were used for amplification of the 16S rRNA gene according to [31], with changes made by adding two nucleotides to the forward primer to increase the annealing temperature. Amplicons (with size around 1500 bp) were separated by electrophoresis in 1.5% agarose gel, stained with ethidium bromide in 0.5 × TBE and analysed using Gel Doc XR+ (Bio-Rad, Hercules, CA, USA). PCR fragments were isolated and purified using the ColGen kit (Syntol, Moscow, Russia), in accordance with the manufacturer’s recommendations. The sequencing of purified PCR fragments was performed using the BigDye Terminator v3.1 cyclic sequencing kit and an automatic DNA Analyzer 3730 sequencer (Thermo Fisher Scientific, Waltham, MA, USA). 

### 2.2. Isolation and Purification of Phage Murka

The Xcc bacteriophage Murka was isolated from a soil sample taken from a field with a massive cabbage black rot outbreak, near Tiraspol (Transnistria, Moldova), in 2012. The phage was propagated using the Xcc Tr1 strain at 26 °C, in accordance with a previously published protocol [32]. Phage lysate was treated with chloroform, and bacterial debris were pelleted by centrifugation at 8000× *g* for 20 min, followed by filtration of the supernatants through 0.22 μm pore-size membrane filters (Millex-GV, Millipore, Cork, Ireland) and the addition of DNAse I (0.5 mg/mL, 1 h; Evrogen, Moscow, Russia). Phage filtrates were concentrated by ultracentrifugation at 100,000× *g* at 4 °C for 2 h, using a Beckman SW28 rotor (Beckman Coulter, Brea, CA, USA). Phage purification was performed by ultracentrifugation in a CsCl step gradient (0.5–1.7 g/mL) at 22,000× *g* for 2 h. The phage-containing opalescent band was collected and dialysed against an SM buffer (10 mM Tris-HCl, pH 7.5, 10 mM MgSO_4_, 100 mM NaCl). The phage suspension was stored at 4 °C.

### 2.3. Electron Microscopy

The morphology of phage particles was studied using transmission electron micros-copy (TEM). Concentrated purified samples of phage Murka were placed on grids and stained with 1% aqueous uranyl acetate (pH 4.0). Prepared grids were examined using a JEM-2100 200 kV transmission electron microscope (JEOL, Tokyo, Japan). The dimensions of each phage were averaged among ~20 individually measured particles.

### 2.4. Biological Characterisation of the Phage Murka

#### 2.4.1. Determination of Phage Host Range on Xcc Strains

To assess the host range among circulating Xcc strains, phage infectivity was tested against the 14 strains listed in Table 1. The set of strains included one reference strain from the NCPPB collection and 13 field isolates. For analysis, 5 µL of phage suspension with a titre of 10^7^ PFU (plaque-forming units)/mL was applied to a double-layer King’s B agar containing the bacterial strain and was incubated overnight at 26 °C. The presence of lytic activity was to be identified by the formation of transparent spots. To exclude false-positive results, phage titration was performed for samples with positive spot tests [33]. 

#### 2.4.2. Phage Adsorption and One-Step Growth Experiments

Adsorption assays were performed according to [34], with some modifications. Strain Xcc Tr1 was grown in King’s B broth at 26 °C to OD_600_~0.2, then infected with phage Murka with a multiplicity of infection (MOI) of 0.1. Every 2–3 min during the early stages of infection, and every 10 min from 20 to 60 min of infection, aliquots of 100 µL of phage were taken and transferred into tubes with 850 µL of SM buffer with 50 µL of chloroform. The mixtures were shaken for 15 min to destroy any remaining bacteria. After bacterial lysis, the mixtures were centrifuged and the supernatant was analysed, to determine the number of unadsorbed, or reversibly bound, phages by plaque assay [34]. The procedure was repeated in triplicate.

One-stage growth assays were performed in accordance with [35]. An exponentially growing culture (10^7^ CFU/mL) of the Xcc Tr1 strain was infected with the phage Murka at an MOI of 0.01. The mixture was then incubated at 26 °C [36] and aliquots of 100 µL were collected every 20 min, cooled to 4 °C, and were centrifuged (8000 rpm, 3 min, 4 °C). Supernatants were titrated using the SM buffer, spread onto King’s B and Tr1 top agar plates, and incubated overnight at 26 °C. The next day, viral plaques were counted. The procedure was then repeated in triplicate and the results were averaged. The latent period was defined as the interval between the adsorption of phages by bacterial cells and the release of the phage progeny. The burst size of the phage Murka was determined as the ratio of the average number of free phage particles after the release phase (plateau average (PFU/mL)) to the corresponding number of phage particles (PFU/mL) added to the exponentially growing bacterial cells.

#### 2.4.3. Phage Stability in Different Conditions

The ability of the phage to survive under various environmental conditions was assessed by incubating a phage sample (10^6^ PFU/mL in SM buffer) at 4, 10, 20, 30, 40, 50, 60, 70, 80, 90, and 100 °C for 1 h in a Thermomixer F 2.0 (Eppendorf, Hamburg, Germany), and adding a series of buffer solutions (20 mM Tris-HCl/20 mM Na-citrate/20 mM Na-phosphate), adjusted with NaOH to a pH in the range of 3–12, to 10^6^ PFU/mL of phage, followed by incubation at 25 °C for 1 h and exposure of the phage sample (10^6^ PFU/mL) in SM buffer under ultraviolet radiation (280–315 nm) using a PL-S9W/12/2p lamp (Philips, Amsterdam, Netherlands), according to [23]. The sensitivity of the phage to chloroform was studied by mixing phage suspensions with different concentrations (5%, 25%, 50%, and 75%) of chloroform and vigorously shaking, followed by incubation at 26 °C for 30 min, according to [37]. The mixtures were centrifuged at 8000 rpm for 15 min and the hydrophilic layer was collected. Phage suspensions were serially diluted with SM buffer and the phage titre was calculated using the double-layer agar method (King’s B agar/Tr1). All tests were carried out in triplicate.

### 2.5. Phage Genome Sequencing and Annotation

Phage DNA was isolated using the standard phenol–chloroform method, after incubation of the sample in 0.5% SDS and 50 μg/mL proteinase K at 65 °C for 20 min. Fragment genome libraries were prepared using 200 ng of genomic DNA as a starting material. DNA was fragmented by ultrasound, using an ME220 focused ultrasonicator (Covaris, Woburn, MA, USA) with the following parameters: iterations—7; duration—10 s; peak power—50; duty factor—20%; cycles per burst—1000. Fragmented DNA was used as an input for library preparation using the NEB Next Ultra II DNA Library Prep Kit for Illumina (New England Biolabs, Ipswich, MA, USA), according to the manufacturer’s instructions. The library was sequenced using a MiSeq sequencer (Illumina, San Diego, CA, USA), using 2 × 250 bp paired-end chemistry, resulting in approximately 435,000 read pairs.

De novo genome assembly was performed using CLC Genomic Workbench 23 (QIAGEN, Aarhus, Denmark). The gene prediction was conducted using Prokka v1.13.4 [38], Glimmer v3.0.2 [39], and Prodigal v2.6.3 [40]. The boundaries of predicted genes were curated manually. The functions of predicted gene products were identified with BLAST [41] and HHpred [42]. The BLAST search employed the NCBI nr/nt databases, and the HHpred search used PDB70_mmcif_2023-06-18, PfamA-v35, UniProt-SwissProt-viral70_3_Nov_2021, and NCBI_Conserved_Domain (CD)_v3.19 databases. The presence of tRNA genes was checked using tRNAscan-SE [43] and ARAGORN [44]. The annotated genome of *Xanthomonas* phage Murka has been deposited in the NCBI GenBank under accession number OR500351.

### 2.6. Genome and Proteome Analysis

Intergenomic comparisons and calculations of intergenomic similarities were performed using clinker [45] and VIRIDIC [46], with default settings. Genetic maps and gene comparisons were visualised using clinker. Protein sequences alignments were made using Clustal Omega [47] and the “number of refinement iterations 3, evaluate full distance matrix for initial guide tree, evaluate full distance matrix for refinement iteration guide tree” command line parameters. Phylogenetic analysis was performed using IQ-TREE v2.2.5 [48] and the “—alrt 1000 -B 5000” command line parameters. The resulting consensus trees with bootstrap support values (1000 replicas) were visualised using iTOL v6 [49]. Protein structures were modelled with AlphaFold 2.2.4 (AF) [50], using full databases and the command line parameter “—monomer” (for a monomeric protein) or “—multimer” (for protein complexes). A proteomic tree was constructed using ViPTree [51] and the built-in dsDNA database.

### 2.7. Statistical Analysis

Data analysis featured the variance method, using Statistica 12.0 (StatSoft, TIBCO, Palo Alto, CA, USA), and comparing the average values using Duncan’s criterion *p* = 0.05. Graphs were plotted using GraphPad Prism 9.2.0.

## 3. Results

### 3.1. Bacterial Strains

**Figure 1:**
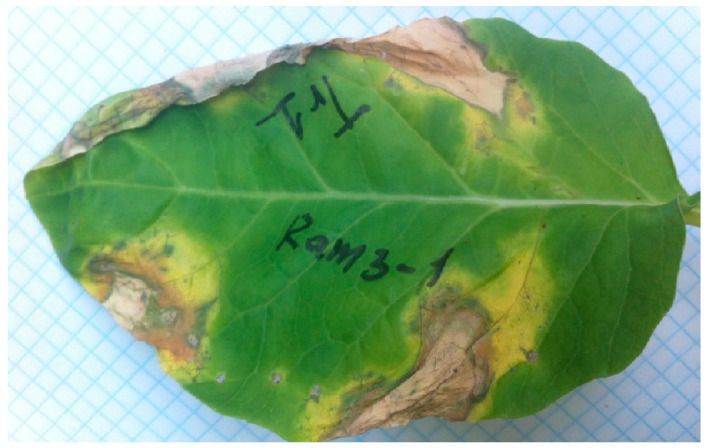
Symptoms of black rot of cauliflower cv. Garantiya infected with *X. campestris* pv. *campestris* Tr1 (upper half of the leaf) and Ram 3-1 (lower half of the leaf) 12 days after inoculation.

The Xcc strain NCPPB 528T was used as a reference for comparison with local Xcc isolates in all experiments [52]. Bacteria were isolated from samples of white cabbage and cauliflower plants with V-shaped chlorotic or necrotic lesions extending from the edges of leaves and blackening of vascular tissues, collected from 2006 to 2017. In total, more than 90 isolates were collected that were phenotypically similar to *Xanthomonas* spp., based on yellow-pigmented mucoid colonies on a YDC nutrient medium. After testing and selection, 13 isolates that belonged to Xcc and the reference strain NCPPB 528T were used in further work (Table 1). All selected strains were: (i) highly virulent for susceptible plants after artificial inoculation (Figure 1); (ii) identical to strain Xcc NCPPB 528T in terms of morphology of the colonies; (iii) positive in the starch hydrolysis test, which is a diagnostic feature of the genus *Xanthomonas* [53]; and (iv) most similar (>95%) in the sequences of 16S rRNA gene fragments to the corresponding sequences of the Xcc reference strains. The annotated 16S rRNA gene sequences of Xcc have been deposited in the NCBI GenBank; the accession numbers are shown in Table 1.

### 3.2. Isolation of Xcc Phage Murka

The Xcc bacteriophage Murka was isolated from a soil sample. The activity of the isolated phage was tested against a number of Xcc strains, resulting in productive infection in 9 of the 14 Xcc strains tested (Table 1). Thus, the phage lysed most strains of the pathogen circulating in Central European Russia, except for one strain from the Moscow region. Tested strains from Belarus, Ukraine, the Netherlands, and the UK were resistant to phage Murka.

### 3.3. Biological Properties of Xcc Phage Murka

#### 3.3.1. Morphology

**Figure 2:**
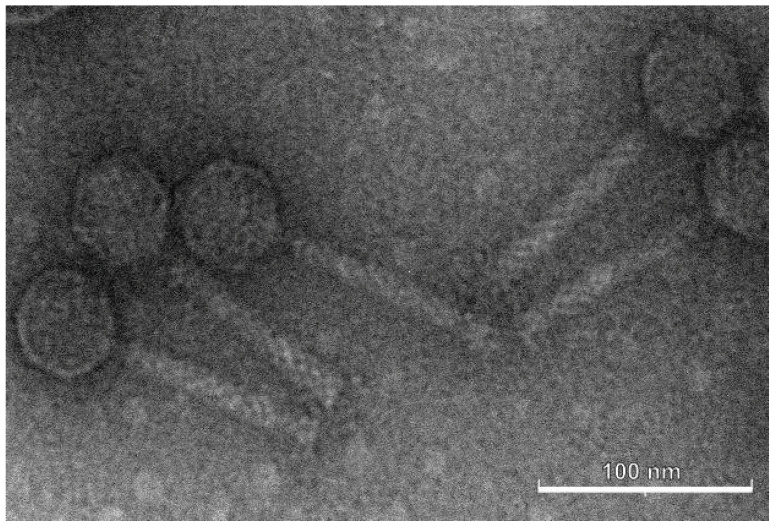
Electron microscopy image of phage Murka. The capsids are icosahedral and ~56 nm in diameter; the tail, about 108 nm long. The scale bar is 100 nm.

Under standard propagation conditions of the bacterial host, Tr1 phage Murka formed small plaques (Ø1–2 mm) with smooth borders and of irregular shape (Supplementary Figure S1). The phage morphology revealed using transmission electron microscopy (Figure 2) demonstrated a typical myovirus appearance; the capsids were icosahedral and ~56 nm in diameter. The tail, about 108 nm long, was connected to the head via a thin neck, and no pronounced fibres/spikes of the adsorption apparatus were observed. 

#### 3.3.2. Phage Production and Stability 

**Figure 3:**
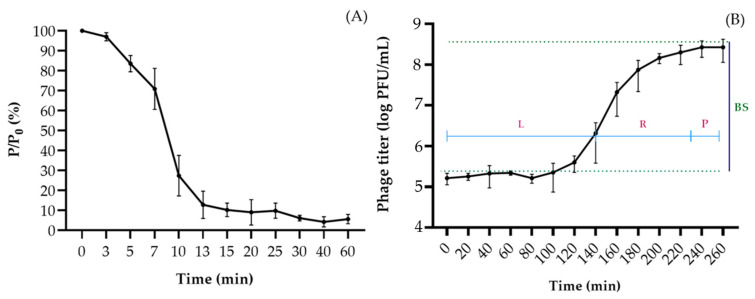
Phage adsorption curve (**A**) and one-step phage growth curve (**B**) of *X. campestris* pv. *campestris* phage Murka. Strain Tr1 was used as a host. The y-axis shows the ratio of the current titre at each time point (P) to the initial one (Po), ×100%. L—latent phase; R—virion release phase; P—plateau phase; BS—burst size. Values in panels represent the mean of three independent trials and error bars represent the standard deviation.

**Figure 4:**
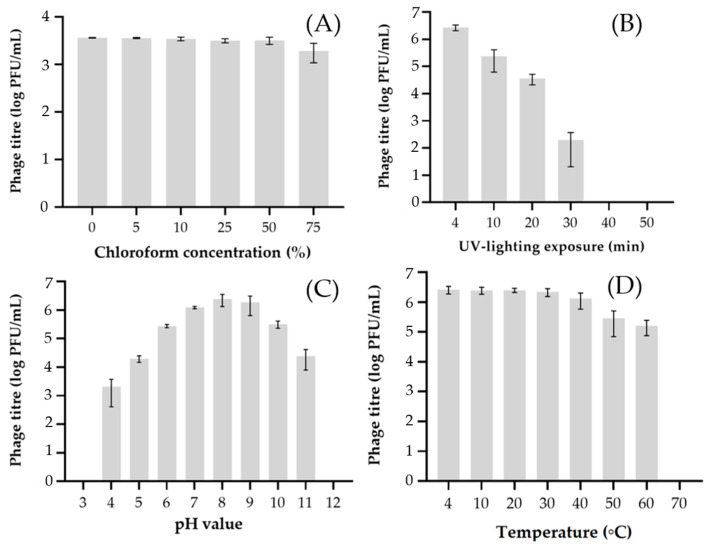
Survival of *X. campestris* pv. *campestris* phage Murka under various stress factors and optimal multiplicity of infection. Phages were mixed with chloroform at a concentration of 5 to 75% (**A**), treated with ultraviolet irradiation for 4 to 50 min (**B**), with a pH from 3 to 12 for 1 h (**C**) and with a temperature from 4 to 100 °C for 1 h (**D**). All tests were repeated three times. The standard deviation (sd) is shown for each bar.

The phage Murka adsorbed to the cells of the host strain Xcc Tr1 almost completely (92.4%) in 13 min (Figure 3A) at 26 °C and lysed the bacteria within 100 min, forming 97 ± 21 progeny particles per infected bacterial cell (Figure 3B). The phage was observed to be resistant to increased concentrations of chloroform: the titre decreased slightly only at 75% concentration in solution (Figure 4A). Ultraviolet (280–315 nm) irradiation reduced phage viability in proportion to the treatment time, with complete destruction after 30 min (Figure 4B). The phage was stable in a pH range of 6–10 at 26 °C for 1 h (Figure 4C), but rapidly lost viability at pH 3–5 and pH 11–12. The infectivity of phage Murka was significantly reduced at temperatures above 50 °C (Figure 4D). In particular, a phage suspension with a concentration of 10^7^ PFU/mL lost 50% of its viability at 50 °C for 1 h. The optimal long-term storage temperature for phages was about 4 °C. 

### 3.4. General Genome and Proteome Features 

*Xanthomonas* phage Murka (GenBank accession #OR500351) has a double-stranded DNA genome of 44,044 base pairs. The GC content of the genome is 59.6% and is distributed evenly throughout the genome’s length. The average GC content of 78 *Xanthomonas campestris* pv. *campestris* contained in the NCBI ReSeq database is 65.1%, which is noticeably higher than the GC content of the phage genome. There are 83 predicted genes in the genome of Murka. Putative functions were assigned to 41 proteins and 42 genes were annotated as encoding hypothetical proteins. No tRNA genes have been found in the genome.

**Figure 5:**
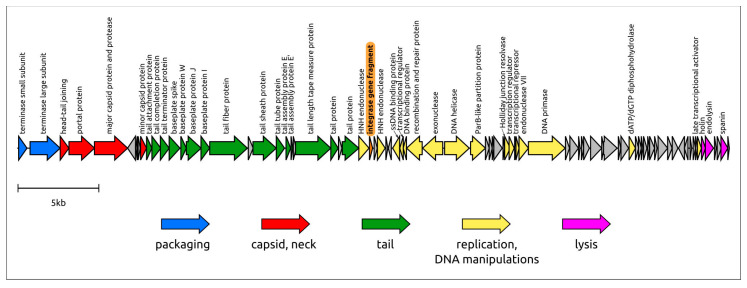
Genetic map of *Xanthomonas* phage Murka. Arrows indicate the direction of transcription. The scale bar indicates the length of the nucleotide sequence. Gene functions are shown in labels and legends. The integrase gene fragment is highlighted orange.

The genome architecture has a modular structure (Figure 5). The structural module contains a block of genes encoding capsid proteins and a block of tail genes. According to the results of AlphaFold modelling, the gene of the major capsid protein (MCP) encodes a major capsid protein with typical HK-97 architecture, protease, and scaffolding proteins as a single 684-amino acid propeptide, as in some other phages [54]. The capsid block also contains a gene of minor capsid protein (mCP). HHpred comparisons and AlphaFold modelling indicate similarities between the mCP of phage Murka and cementing proteins with jellyroll topology [55].

The tail block comprises 18 predicted genes involved in tail assembly. The tail sheath protein (TShP) that forms the contractile sheath around the tail tube in myoviruses and type VI secretion systems contains a core domain and an additional β-barrel domain, like some phages that are not large in size [56]; this structural architecture is characteristic for all *Foxunavirus* phages. Apparently, the functions of the receptor-binding protein (RBP) are performed by the tail fibre protein (TFP). The TFP of phage Murka contains an *N*-terminal part that is reminiscent of the TFP of phage P1, a temperate Myophage [57], and a triple-β-helix that is possibly responsible for binding to the receptor. Lipopolysaccharides have been suggested to be the primary receptors for *Foxunavirus* phages [22].

About half of Murka’s genome contains genes involved in replication and DNA metabolism. The replication apparatus contains none of its own DNA polymerase (DNAP). Probably, phage Murka uses host DNAP, such as phage λ [58], but the genome encodes its own DNA helicase and DNA primase. 

The phage genome contains a gene encoding a small (64 aa) C-terminal fragment of integrase. According to sequence comparisons (Supplementary Figure S2) and HHpred analysis, the hypothetical protein encoded by this gene accounts for only about 10–20% of average phage integrase. An integrase-like gene fragment and a small orphan gene downstream this fragment gene are located adjacent to the HNH endonuclease sequences. Genes involved in lysogeny decisions were not detected by either BLAST or remote homology searches.

The Murka’s lysis machinery appears to mediate a three-step lysis that is typical of tailed phages [59]. The genome lysis block includes genes encoding holin, endolysin, and spanin.

### 3.5. Intergenomic Comparisons and Phylogenetic Analysis

#### 3.5.1. Comparisons with Related Phages

**Figure 6:**
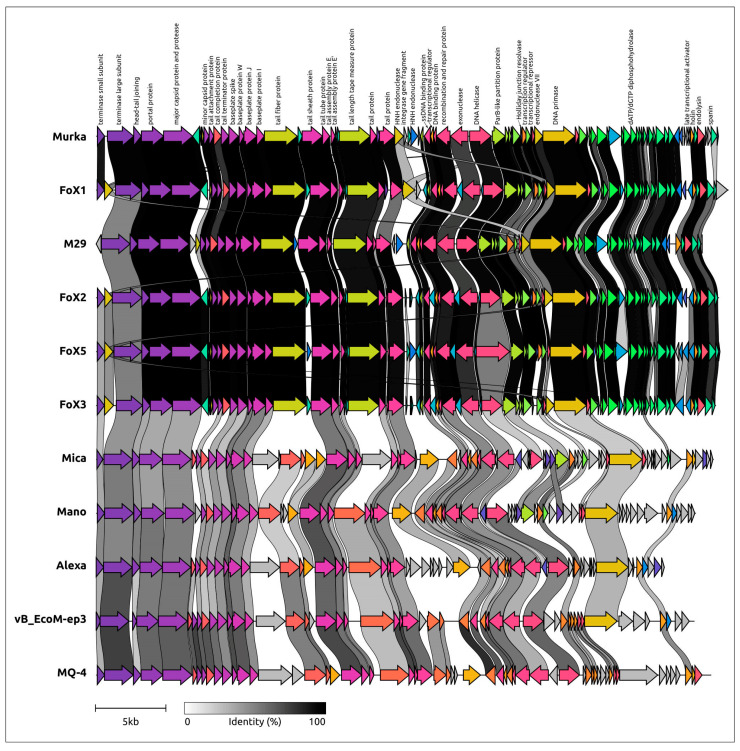
Comparative genome alignment of *Xanthomonas* phage Murka and related phages *Xanthomonas* phage FoX1 (FoX1), *Xanthomonas* phage M29 (M29), *Xanthomonas* phage FoX2 (FoX2), *Xanthomonas* phage FoX5 (FoX5), *Xanthomonas* phage FoX3 (FoX3), *Burkholderia* phage Mica (Mica), *Achromobacter* phage Mano (Mano), *Acinetobacter* phage Alexa (Alexa), *Escherichia* phage vB_EcoM-ep3 (vB_EcoM-ep3), and *Serratia* phage MQ-4 (MQ-4). The percentage of amino acid identity is represented by greyscale links between genomes. Homologous proteins are assigned a unique colour.

A BLAST search using sequences of Murka’s sequences indicated that *Foxunavirus* phages were the closest relatives. The search also found related phages infecting bacteria, other than *Xanthomonas*, including *Burkholderia* phage Mica (genus *Micavirus*) [60], *Achromobacter* phage Mano (genus *Manovirus*) [61], *Acinetobacter* phage Alexa (unclassified), *Escherichia* phage vB_EcoM-ep3 (genus *Jilinvirus*) [62], and *Serratia* phage MQ-4 (unclassified) [63]. The results of comparative genome alignment showed a similarity of genomic organization between these phages, *Foxunavirus* phages, and phage Murka (Figure 6). Interestingly, phage Alexa contains a gene that apparently encodes a functional integrase (length 397 aa), and phages Mano, Mica, and MQ-4 also have integrase genes encoding proteins 403–445 aa long.

#### 3.5.2. VIRIDIC Analysis 

**Figure 7:**
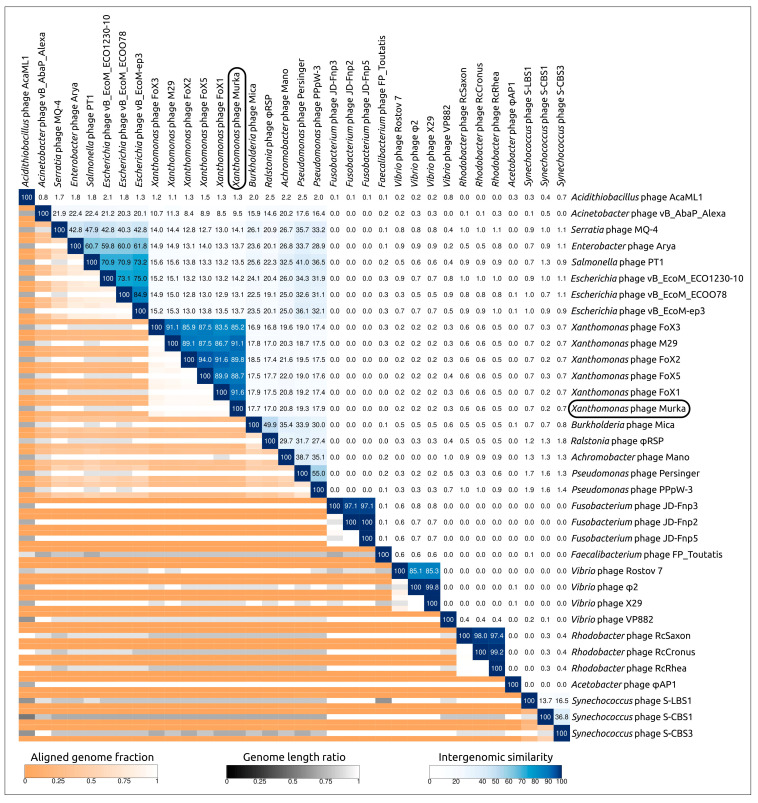
VIRIDIC-generated heatmap of *Xanthomonas* phage Murka and related phages. The colour coding in the upper right part of the map indicates the clustering of the phage genomes based on intergenomic similarity. The numbers represent similarity values for each genome pair, rounded to the first decimal. The aligned genome fraction and genome length ratio are shown in the lower left of the map, using a colour gradient in the legends.

Calculations of nucleotide-based intergenomic similarities were conducted using VIRIDIC. This tool is recommended by ICTV as a primary classification technique [46]. VIRIDIC analysis was performed using genomic sequences of related phages found through BLAST searches using Murka protein sequences as queries. The analysis indicated a high level of intergenomic similarity (91.6%) between *Xanthomonas* phage Murka and the closest phage FoX1 (*Foxunavirus fox1*) (Figure 7), which is lower than the 95% species delimitation threshold and higher than the 70% genus delimitation threshold. According to this analysis, the phage Murka clusters with previously reported *Foxunavirus* phages, with an intergenomic similarity of 83.5–94.0% within the corresponding cluster. Since the threshold for regular genus classification is 70%, the results of the VIRIDIC analysis enable phage Murka to be classified as a new species within the genus *Foxunavirus*. In addition, convincing similarity between the proteomes of phage Murka and *Foxunavirus* phages was indicated with a ViP proteomic tree (Supplementary Figure S3). 

#### 3.5.3. Phylogenetic Analysis 

**Figure 8:**
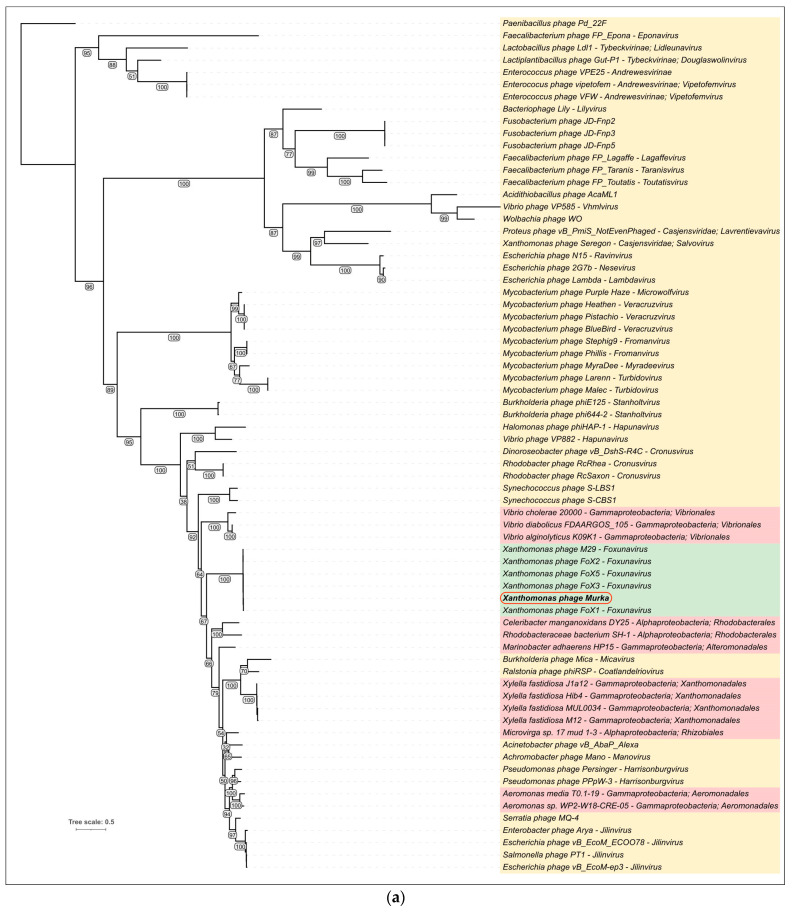

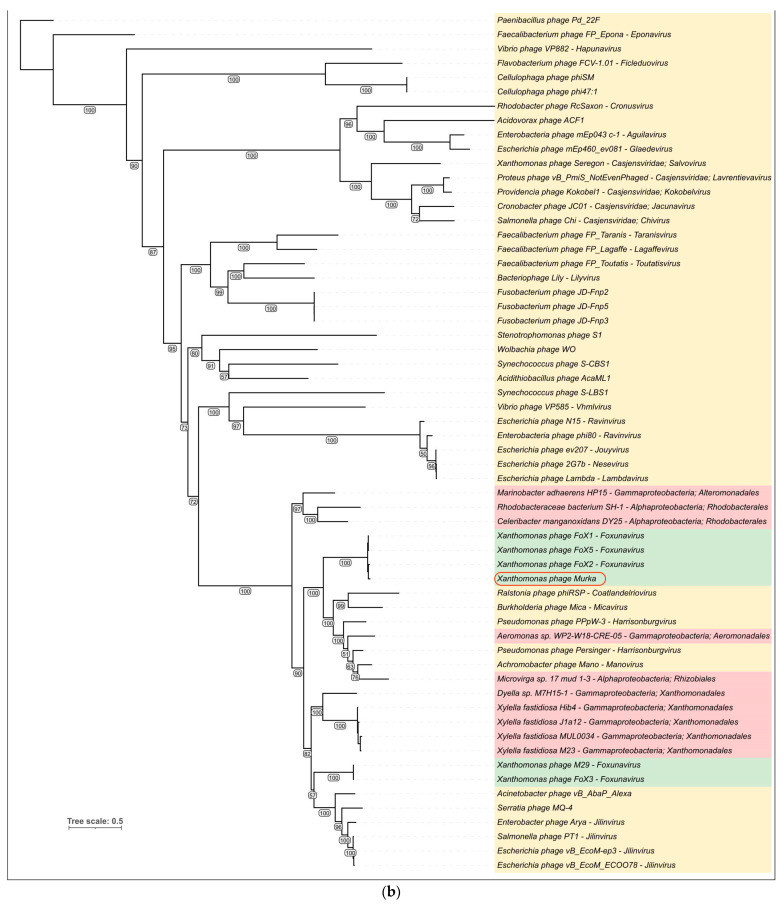
Phylogenetic trees based on amino acid sequences of MCP (**a**) and TLS (**b**). *Foxunavirus* phages are highlighted in green, phage sequences of other taxa are highlighted in yellow, and bacterial sequences are highlighted in red. Bootstrap values are shown near their branches. Branches with a bootstrap support lower than 50% were deleted. The scale bar shows 0.5 estimated substitutions per site, and the trees were rooted to *Paenibacillus* phage Pd_22F.

Phylogenetic analysis was performed using closely related amino acid sequences of the major capsid proteins (MCP) and terminase large subunit (TLS) found in phage genomes and bacterial prophage sequences (Figure 8). The construction of phylogenetic trees used the sequences of MCP and TLS found not only in phage genomes, but also in putative homologous genes found in prophage regions of bacterial genomes. Homologues of MCP and TLS of the phage Murka have not been identified in *Xanthomonas campestris* pv. *campestris* but have been found in the genomes of other bacteria, and this may suggest the evolutionary traits of *Foxunaviruses*. Interestingly, the topology of MCP and TLS trees is different, which may be a consequence of gene exchanges resulting in the modular evolution of phage genomes. In particular, the TLSs of *Xanthomonas* phages FoX3 and M29 cluster differently than the TLSs of other *Foxunavirus* phages.

## 4. Discussion

Black rot of cabbage caused by Xcc is one of the most notorious bacterial diseases of plants, leading to losses of yield and quality in cabbage crops. Currently, disease control is complex and combines the principles of integrated plant protection [18]. Following the trend to reduce the use of pesticides in the production of crop products and the need to improve product quality, the task is to research and implement new methods of plant protection. Recently, alternative methods proposed for Xcc control have included the use of resistance inducers together with antagonist bacteria [64], botanical pesticides [65], and RNA interference [66]. 

As can be seen in recent publications, the interest in the use of phage therapy in agriculture is increasing [67]. Reviewed research on phage-based biocontrol of plant pathogens is targeting the most common and destructive plant pathogens and includes some Xcc trials [22]. A number of studies have shown that the use of phages to control bacterial plant diseases is a promising option, because they are cheap to produce, self-destructible, and safe for the host organism and the biosphere, since they are a natural part of it [68,69]. An undeniable advantage of phages in comparison with other agents for the control of bacterial diseases is the possibility of their use to control synthetic biocide-resistant forms of bacteria [70]. This is an important point in the case of *Xanthomonas* spp., whose horizontal gene transfer (HGT) is known to play an important role in the development of resistance [70]. HGT is known to play a key role in the evolution of pathogenic bacteria, so it has been described that almost 60% of *Xanthomonas* genes were acquired by HGT [71].

Due to the meagre effectiveness of chemical plant protection methods in the control of bacterial diseases, the environmental problems with their use, and the emergence of resistant forms of phytopathogenic bacteria, phages provide an alternative means of combatting Xcc [72]. In this study, a new Xcc phage, Murka, was isolated, and a characterisation was carried out to reveal its effect on bacteria.

The phage was isolated from the soil in which cabbages infected with Xcc were cultivated. Similar to Murka, a number of Xcc phages have been isolated from soil from fields with symptomatic plants [72,73,74]. This may indicate that phages can survive only in an environment with a large supply of host biomass, since they are obligate parasites of bacteria. 

Interestingly, the phage lysed strains from the Krasnodar and Moscow regions in Russia, as well as those from the city of Tiraspol (Moldova) and Japan, while strains from Ukraine, Belarus, the Netherlands, the UK, and one strain from Dmitrov (in the Moscow region, Russia) were not subject to lysis by the phage. This finding may reflect the differences in receptor determinants in host strains of different geographical origin, and the phage reaction may facilitate the assessment of the diversity of Xcc strains across the world.

According to the results of intergenomic comparisons, phage Murka should be classified as a member of the genus *Foxunavirus*. Closely related phages FoX1, FoX2, FoX3, and FoX5 seem to adhere to a strictly lytic infection cycle [22]. It is worth noting that bioinformatic and phylogenetic analysis showed the genomes of phage Murka and other *Foxunavirus* phages to be similar to the genomes of phages having a lysogeny apparatus, which were presumably capable of a temperate lifestyle. The putative integrase pseudogene surrounded by homing endonuclease genes in the genome of Murka is, however, unlikely to encode a functional integrase. Similar “remnants” of integrases were detected in the genome of some phages more distantly related to Murka and other Foxunaviruses. An attempt was made to trace the hallmark genes of *Foxunavirus* phages in prophage regions of the genomes of *Xanthomonas* spp., but no homologues of genes encoding a major capsid protein and terminase of phage Murka were found. Therefore, it can be assumed that the gene of functional phage integrase of *Foxunavirus* predecessors was destroyed by mobile elements, which led to the transition to a lytic lifestyle. Eventually, this transition occurred before the host switch to *Xanthomonas*. A relatively recent host switch can be suggested from the noticeable difference in GC content between the genomes of *Xanthomonas* and *Foxunavirus*.

Phage Murka has a latency period of ~140 min, followed by a virion release phase of 140 to 230 min, with a burst size of 97 virions per bacterial cell. These values are more favourable for phage control purposes than those typical of some other previously characterised Xcc phages [73,75]. 

Thus, the phage has been shown to be a promising possible agent of Xcc control. Undoubtedly, this study is primary and in the future we are planning to evaluate the effect of this phage on the parameters of black rot development on cabbage plants in an artificial inoculation, to assess the applicability of preparative forms of phage solution and to determine the optimal method of phage application on cabbage plants.

## 5. Conclusions

A new lytic phage, Murka, which lyses 64% of the Xcc strains used in the study, has been characterised. It has a typical myovirus morphology and belongs to *Foxunavirus*. This phage is a promising agent for the biological control of the black rot of cabbage. In order to prove this, it is necessary to carry out the tests on plants under artificial infestation conditions.

## Figures and Tables

**Table 1:** The spectrum of lytic activity of the phage Murka against *X. campestris* pv. *campestris*.

Strain Number	Name of the Strain	Date of Isolation	Place of Origin, Plant	Genbank 16S №	Lysis Zone during Interaction with the Phage Murka
1	BK-55	10.2017	Krasnodar region, Russia, white cabbage	OR626094	+
2	CK-71	10.2017	Krasnodar region, Russia, cauliflower	OR626097	+
3	Xcc 1/1	09.2017	Moscow region, Dmitrov district, Russia, white cabbage	OR626648	+
4	Bes-1	09.2016	Moscow region, Dmitrov district, Russia, white cabbage	OR626092	+
5	Cas	09.2016	Moscow region, Dmitrov district, Russia, cauliflower	OR626095	+
6	Tr1	11.2012	Tiraspol, Moldova, cabbage	OR626099	+
7	DK-1	10.2012	Moscow region, Serpukhov district, Russia, white cabbage	OR626096	+
8	Ram 3-1	10.2012	Moscow region, Ramensky district, Russia, cabbage	OR625211	+
9	XУ 1-2	10.2012	Ukraine, white cabbage	OR644606	-
10	Bel-2	10.2006	Belarus, white cabbage	OR626091	-
11	Bun-1	09.2006	Moscow region, Dmitrov district, Russia, white cabbage	OR626093	-
12	Xn-13	1997	Japan, Ano, *Capsélla búrsa-pastóris* (shepherd’s purse)	OR626098	+
13	306NZ	-	Netherlands	OR626090	-
14	NCPPB 528T	1957	UK, cabbage	-	-

## Data Availability

Data is contained within the article or Supplementary Material.

## Supplementary Materials

The following supporting information can be downloaded at: https://www.mdpi.com/article/10.3390/v16020198/s1, Figure S1. The morphology of Murka phage plaques on 0.7% upper YD agar with a strain of the host bacterium Tr1. A total of 10 µL of phage suspension was poured into 3.5 mL of upper agar with the bacterium, distributed on a dish with lower YD and observed for 24 h after cultivation at 26 °C. A—general view of the dish, B—magnification of a separate area. Figure S2. The alignment of protein gp30 and integrase of *Achromobacter* phage Mano. Pfam domains are shown in purple. Figure S3. ViP proteomic tree constructed based on predicted proteomes of phage Murka and dsDNA phages.

## Abbreviations

Xcc	*Xanthomonas campestris* pv. *campestris*
cv.	Cultivar
CFU	Colony-forming unit
PFU	Plaque-forming unit
IKI	Solution of molecular iodine (I) and potassium iodide (KI)
MOI	Multiplicity of infection
OD	Optical density
AUGC	Area under growth curve
