# Supplementary figures and images for: Characterisation of New Foxunavirus Phage Murka with the Potential of Xanthomonas campestris pv. campestris Control
Source: Viruses. 2024 Jan 27;16(2):198. doi: 10.3390/v16020198 (PMC10892653; doi:10.3390/v16020198)

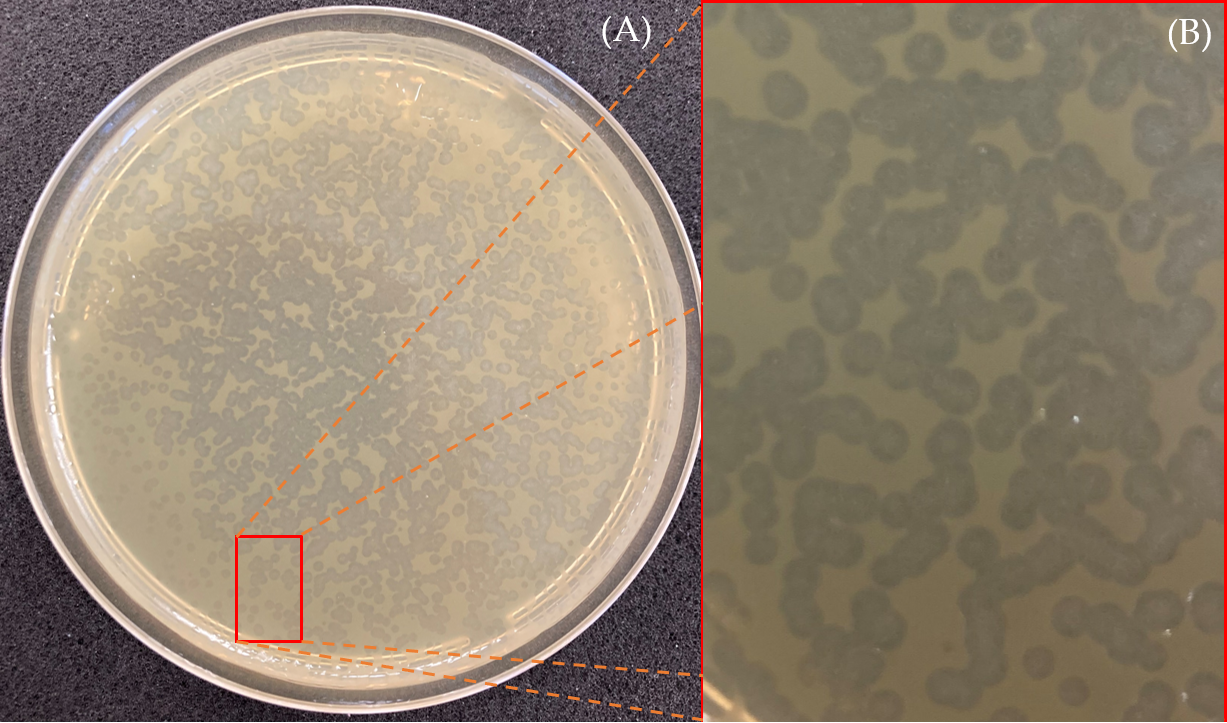

Supplement: Supplementary file 1 [file viruses-16-00198-s001.zip › Suppl_Fig_S1.png]

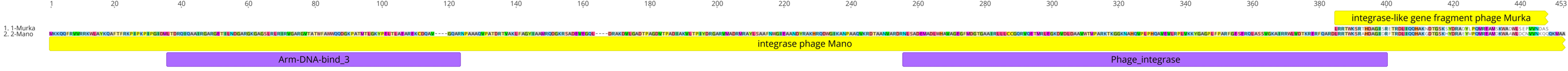

Supplement: Supplementary file 1 [file viruses-16-00198-s001.zip › Suppl_Fig_S2.jpg]

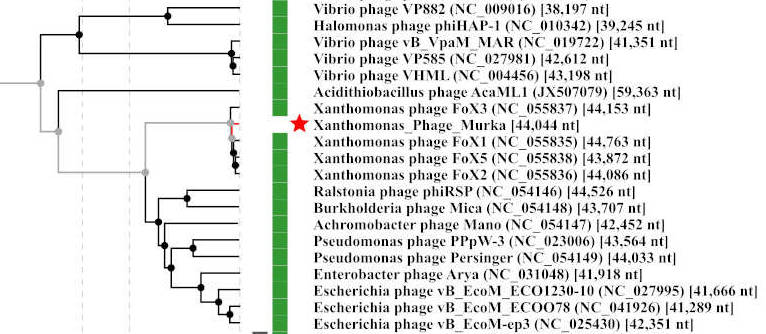

Supplement: Supplementary file 1 [file viruses-16-00198-s001.zip › Suppl_Fig_S3.jpg]
